# Fungal Community Composition as Affected by Litter Chemistry and Weather during Four Years of Litter Decomposition in Rainshadow Coastal Douglas-Fir Forests

**DOI:** 10.3390/jof8070735

**Published:** 2022-07-16

**Authors:** Philip-Edouard Shay, Richard S. Winder, C. Peter Constabel, J. A. (Tony) Trofymow

**Affiliations:** 1 Centre for Forest Biology, Department of Biology, University of Victoria, Victoria, BC V8W 3N5, Canada; philip-edouard.shay@nrcan-rncan.gc.ca (P.-E.S.); cpc@uvic.ca (C.P.C.); 2 Pacific Forestry Centre, Canadian Forest Service, 506 West Burnside Road, Victoria, BC V8Z 1M5, Canada; richard.winder@nrcan-rncan.gc.ca

**Keywords:** carbon, climate change, proanthocyanidins, in situ decay, *Pseudotsuga menziesii*, *Populus angustifolia*

## Abstract

Climate and litter chemistry are major factors influencing litter decay, a process mediated by microbes, such as fungi, nitrogen-fixing bacteria and ammonia-oxidizing bacteria. Increasing atmospheric CO_2_ concentrations can decrease nitrogen (N) and increase condensed tannin (CT) content in foliar litter, reducing litter quality and slowing decomposition. We hypothesized that reduced litter quality inhibits microbes and is the mechanism causing decomposition to slow. Litterbags of Douglas-fir needles and poplar leaves with a range of N (0.61–1.57%) and CT (2.1–29.1%) treatment and natural acid unhydrolyzable residue (35.3–41.5%) concentrations were placed along climatic gradients in mature Douglas-fir stands of coastal British Columbia rainshadow forests. The structure (diversity, richness and evenness) and composition of microbial communities were analyzed using DGGE profiles of 18S, NifH-universal and AmoA PCR amplicons in foliar litter after 7, 12, 24 and 43 months of decay. High CT and low N concentrations in leaf litter were associated with changes in microbial community composition, especially fungi. Contrary to our hypothesis, high CT and low N treatments did not inhibit microbial colonization or diversity. The joint effects of air temperature and soil moisture on microbial community composition at our sites were more important than the effects of initial litter chemistry.

## 1. Introduction

Decaying litter is one of the largest terrestrial sources of CO_2_ released into the atmosphere [1], impacting carbon sequestration and nutrient-cycling regimes. Litter decomposition is a process mediated by microbial communities and primarily regulated by macro- and microclimate, substrate quality, litter size, microbiome diversity and exogenous nutrient availability [2,3,4]. Climate change resulting from ongoing anthropogenic CO_2_ emissions is predicted to lead to a generally warmer global climate and altered moisture regimes [5] increasing decay rates [6,7] via increased microbial metabolism [1,6] and altered microbial competitive landscapes [7]. The full extent of potential climate change impacts on soil microbial communities is undetermined [8]. Faster decomposition in northern forests could release vast quantities of carbon (C) now stored in soils and decaying plant matter. For example, up to 70.6Gt could be released from Canadian forests that would exacerbate climate change [9,10].

Greater atmospheric CO_2_ concentrations are predicted to increase condensed tannins (CTs) and acid unhydrolyzable residues (AURs) and decrease nitrogen (N) concentrations in foliage of woody plants [11,12,13,14]. Higher CT and AUR concentrations in litter have been shown to slow the decomposition of foliage [15,16,17,18,19] and N mineralization [3,20]. These cascading effects could impact associated microbial communities, but these have not been extensively investigated. The biosynthesis of CTs is under genetic as well as environmental control [21], leading to a wide range of CTs differing in polymer length, B-ring hydroxylation and stereochemistry [22]. Variation in CT structure is associated with altered protein-binding and oxidative properties [23], which can impact below-ground microbial communities in multiple ways. CTs can inhibit microbial growth via direct toxicity [24] or via complexation with substrates such as cell wall polysaccharides [25], nitrogenous compounds [16,26] and exoenzymes [27].

Three microbial functional groups play especially important roles in decomposition and nutrient cycling in temperate forests, viz. fungi, nitrogen-fixing bacteria and ammonia-oxidizing bacteria. Fungal communities are the most abundant microbes in soils of conifer forests and play an important role in litter decay and nutrient cycling [28]. A variety of saprophytic and mycorrhizal fungal species are able to decay litter using extracellular enzymes [29] and are biogeographically distributed according to climate, substrate quality, vegetation [30] and in some cases dispersal limitations [31,32]. Nitrogen is limited in temperate forests [33], but essential for meeting the stoichiometric requirements of microbes responsible for decay [34]. Free-living diazotrophs are important sources of N input into forest ecosystems [33,35] and can be responsible for exogenous N sources in litter with low N availability, thus influencing decay rates. By catalyzing the rate-limiting step for nitrification, ammonia-oxidizing bacteria act to increase NH_3_ availability [36]. Fungi are affected by CT amendments in diverse experimental systems [37]. Free-living diazotrophs are sensitive to environmental disturbance [38], and they also react to CT amendments in experimental microcosms [37].

In an effort to quantify the effects of litter chemistry and climate on litter decomposition and N mineralization, we designed a field study that used bags of leaf litter with different levels of CTs and N to assess the impact of these variables on in situ decay rates and associated microbes in the forest floor over a period of 43 months. This study spanned multiple field sites located along a temperature and moisture climatic gradient, each site composed of similar, typical over- and understory species dominated by mature Douglas-firs. Shifts in plant species along climate gradients can influence microbial composition in soils and confound field analyses of microbial responses to climate [39]. In this study, the similarity of vegetation along the climate gradient was ideal for assessing the impacts of climate on decay-associated microbes in the field. Furthermore, previously established biogeographical assessment of microbial communities in the forest floor and mineral soil at these sites [40] provided a unique baseline from which to compare microbial responses observed on decaying leaf litter.

Our particular objectives were to assess how the structure (diversity, richness, evenness) and composition of microbial functional groups associated with decaying litter might be affected by litter chemistry and climate gradients. This included assessment of temporal and spatial components, since microbial communities are known to shift with time [41] and space as litter decay proceeds [42]. Polymerase chain reaction coupled with denaturing gradient gel electrophoresis (PCR-DGGE) was used as a cost-effective technique for microbial community fingerprinting. PCR-DGGE is sufficient for assessment of community structure and diversity when functional groups are targeted, allowing for basic screening and supporting focused analysis of treatment effects on abundant (>0.5% relative abundance) functional groups [43,44,45].

In the present study, we tested the hypotheses that (1) slower decay rates associated with high CT levels and low N concentrations in leaf litter are caused by inhibition of microbial colonization and diversity and that (2) initial litter chemistry continued to be an important determinant of microbial community composition over 43 months of litter decay.

## 2. Methods

### 2.1. Litter Bags and Field Sites

To assess decay impacts in forest floor soils, leaf litter samples having a range of N (6–15 mg g^−1^) and CT (21–278 mg g^−1^; Table 1) levels were placed in fine, polypropylene mesh (0.5 mm opening) litter bags (25 × 25 cm) for decay on the forest floor, as described in [19,46]. Using litter-fall traps, naturally abscised leaf litter from *Populus angustifolia* (poplar) and *Pseudotsuga menziesii* (fir = Douglas-fir) was obtained in 2008 from a common garden established in 1991 at the Ogden Nature Center (Ogden, UT, USA) and in 1991 from the Shawnigan Research Forest [46], respectively, and oven-dried (50 °C) prior to sealed storage in darkness. Litter bags were filled with either 5 g of bulked fir needles or 4.6 g of poplar leaf litter (oven-dried weight, 12 h at 70 °C). High and low CT treatments for poplar litter were made by bulking leaves from clones with known CT level, while ensuring other nutrients (e.g., N) and phenols (e.g., salicortin) were kept constant between treatments [47,48]. High-N litter treatments were prepared by spraying an atomized glutamine solution onto individual leaf-litter samples, while control (low-N) treatments were sprayed with an equal amount of distilled H_2_O. Determination of C, N, CT and proximate chemistry (e.g., AUR) in leaf litter prior to decay was outlined in [19,49].

Four strings of litter bags, each with six litter types (Table 1), were laid on the forest floor at four replicated microplots (ca. 2.5 × 2 m) in close proximity (<23 m) to meteorological stations at nine sites on southeastern Vancouver Island, BC, Canada [40]. Sites were located along south, central and north transects spanning the coastal Douglas-fir biogeoclimatic (BGC) zone (DF), coastal western hemlock biogeoclimatic zone (WH) and the transition between the two (TR) [50]. Moisture probes and thermocouples connected to CR10 (Campbell Scientific, Logan, UT, USA), HOBO (models U30 and U12, Onset Computer Corporation, Bourne, MA, USA) and Decagon (model Em50 Digital, Decagon Devices, Pullman, WA, USA) data loggers and Ibuttons (Maxim Integrated, San Jose, CA, USA) recorded hourly measurements of air and soil temperature (30 cm above and below ground, respectively), along with soil moisture at each site.

### 2.2. Sample Collection and Processing

Litter bags were collected after 7, 12, 24 and 43 months of decay in the field, and kept cool (4 °C) until processed in the laboratory within 36 h after removal. For microbial analysis, a representative subsample (0.25 g wet weight) of each litter sample was removed from litter bags using sterile forceps and gloves; the remaining litter fraction was used to assess % C mass remaining and N mineralization [19]. For each sampling period, four replicate subsamples of each litter treatment were pooled by site and homogenized manually using sterile technique. The pooled subsamples were stored at −20 °C prior to DNA extraction. For extraction, samples were first pulverized (1 min at 30 Hz) using a liquid N compatible bead beater (Retsch Mixer Mill MM400, Newtown, PA, USA). DNA was extracted from 0.25 g of pulverized tissue using PowerLyser^TM^ PowerSoil DNA Isolation kits (according to manufacturer’s protocol and including a 10 min incubation period at 70 °C for optimal cell lysis). Extracted DNA was assessed for quantity (μg/mL) and quality (λ absorbance ratios 230/280 and 230/260) using an ND-1000 spectrophotometer (NanoDrop products, Thermo Fisher Scientific, Wilmington, DE, USA) to account for amplification bias that could occur during DNA extraction.

Primer sets for amplification of microbial functional groups each contained GC-clamps in order to prevent complete separation of complementary strands in PCR products during DGGE [40,51,52]. PCR-DGGE fingerprinting of fungi, ammonia-oxidizing β-proteobacteria and free-living diazotrophs was performed using primer sets *18S* FF390/FR1, *AmoA* 1F’/2R, and *NifH*-universal ForA/ForB/Rev, respectively, according to modifications by [40] of existing protocols [53,54,55,56].

Pooled DNA from all samples in each sampling period was used as standard ladders to allow for band comparison within and between gels, and PCR runs (see Figure S1 from [40]). The migration distances of bands were determined using GeneTools software (Syngene, Frederick, MD, USA) and consolidated from at least two reference ladders per gel, followed by visual inspection. Operational taxonomic units (OTUs) obtained from PCR-DGGE gels were consolidated into a presence/absence matrix across time and space, while values for the sum of all peak heights for all bands in a lane (*N),* the total number of bands in a lane (*S*) and the peak height of the *i^th^* DGGE band in a lane (*n_i_)* were used for calculating diversity indices (Shannon’s diversity—Equation (1), richness—Equation (2) and Pielou’s evenness—Equation (3)) for each sample.

(1) $$H^{\prime }=-\sum \left[\frac{n_{i}}{N}\right]log\left[\frac{n_{i}}{N}\right]$$

(2) $$\;d=\frac{\left[S-1\right]}{log\left[N\right]}$$

(3) $$\;J=\frac{H^{\prime }}{log\left[S\right]}$$

### 2.3. Data Analysis

R Statistics software (version 3.3.1) was used for all statistical analyses [57]. We first modeled the multivariate response of DNA extraction efficiency (DNA concentration, 230/280 λ absorbance ratio and 230/260 λ absorbance ratio) to time, latitude, zone, litter type and their interactions, using constrained redundancy analyses (RDAs), followed by post hoc analysis of each trait using univariate regression. The effects of climate (Table 2), litter type (Table 1), time and space on microbial communities associated with decaying litter were determined with partially constrained RDA and correspondence analysis (CCA) [58,59] using the R package ‘vegan’ [60]. Climate, litter chemistry and time were each assessed as categorical (e.g., north DF, central DF and south DF) and continuous (e.g., mean, minimum and maximum annual air temperature) predictors (Table 1 and Table 2).

Categorical modeling of climatic effects was assessed by site (i.e., nine unique latitude and zone combinations), since latitude and zone did not always reflect clear climatic gradients. However, site categories represented both climatic and spatial effects, which were separated only in models using continuous predictors where spatial factors were determined by principal coordinates of neighbor matrices (PCNMs) [61]. Similar to methods first described to parse out spatial variation [58,59], microbial community responses were first assessed separately for time, climate, litter chemistry and PCNM effects, then jointly in partially constrained models, in order to determine the proportion of shared effects. Categorical models were also assessed after removing any significant effects of DNA extraction efficiency. The significance of models and individual predictors was assessed by permutation tests for RDA and CCA using reduced models.

Categorical models were reduced by step-wise elimination of least significant predictors. In continuous models, the number of climate and PCNM predictors (and litter chemistry predictors in the case of joint poplar and fir analyses) was reduced using step-wise model building according to AIC-like criteria [60]. This was followed by elimination of non-significant factors while assessing each separately. Candidate climate variables only included those with Pearson’s correlation *r* < 0.81 (Table 2). Elemental composition, proximate chemistry and CT variables were included as litter chemistry effects when assessing both poplar and fir data sets jointly, but only N and CT (poplar only) content were considered when assessing poplar and fir data sets separately. Post hoc analysis of litter chemistry effects in poplar litter was also performed on reduced data sets, removing the 43-month data. When considering all three targeted functional groups, best fit models were also confirmed using ‘leap’ model selection for multivariate responses [62]. In each case, models and predictors were deemed significant by permutation tests.

Responses in microbial community structure (Shannon’s diversity, richness and Pielou’s eveness) or composition (OTU presence or absence) of fungi, ammonia-oxidizing bacteria and nitrogen-fixing bacteria were assessed jointly and also separately for each functional group. Data sets for poplar and fir litter types were also treated jointly and separately. When modeling microbial structure, responses were assessed for multivariate normality, power transformed where appropriate and missing data (OTU absence causing null measurements) were imputed using the iterative PCA algorithm from the ‘missMDA’ R package in order to reduce the influence of such values in ordination analyses [63]. The effects of % C remaining in litter samples on overall microbial responses were also assessed with and without partial constraint by best-fit predictors. Results on the %C mass remaining in different litter treatments over 43 months have been previously published [19].

## 3. Results

Fingerprinting of microbial functional groups using PCR-DGGE identified 66 unique fungal OTUs, 23 *NifH* OTUs and 23 *AmoA* OTUs distributed among 192 samples.

### 3.1. DNA Quality

DNA quality from poplar litter was affected by time, site and litter type (*p* = 0.001, 0.013 and 0.009, respectively), specifically due to generally greater DNA concentration over time and in LTHN treatments, lower 260/280 λ ratios over time and in the SDF site and greater 260/230 ratios in 43-month samples (post hoc *p* < 0.05). DNA quality in fir litter was also affected by time (*p* = 0.001), due to a significant decrease in the 260/280 λ ratio over time of decay (*p* = 0.004). The 260/230 λ ratios in fir samples decaying for 12 and 24 months were on average half the values in samples at 7 and 43 months (post hoc *p* < 0.001). DNA concentrations increased in fir samples until 24 months of decay followed by a drop to 12-month levels (post hoc *p* < 0.019). On average, DNA concentration was 23.4% less in poplar than fir litter (*p* = 0.002), while the 260/280 λ ratio was 12.8% greater (*p* < 0.001). However, DNA quality generally did not confound significance of categorical models (Table 3). Only changes in fungal community composition associated with N treatments in fir litter, which were not significant when using continuous variables, could instead have been attributed to DNA concentration effects (Table 3B).

### 3.2. Temporal Effects

**Figure 1:**
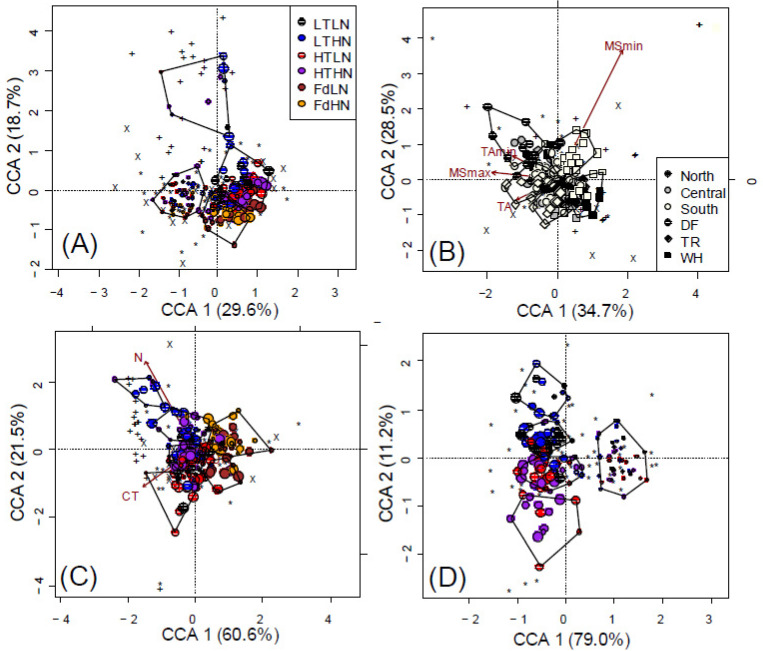
Constrained correspondence analysis showing the effects of (**A**) time, litter chemistry, climate and space (14.8% of total inertia) on microbial composition of fungi (*), *NifH* (+) and *AmoA* (X) communities. Partially constrained correspondence analysis (**B**) removing all effects except climate (2.75% of total inertia) or (**C**) litter chemistry (3.2% of total inertia) is also shown. The effects of both time and litter chemistry (**D**) on only fungal community composition in poplar litter (7.6% of total inertia). Point size decreases with time in panels A, C and D. Polygons represent k-means clustering based on CCAs 1 and 2.

**Figure 2:**
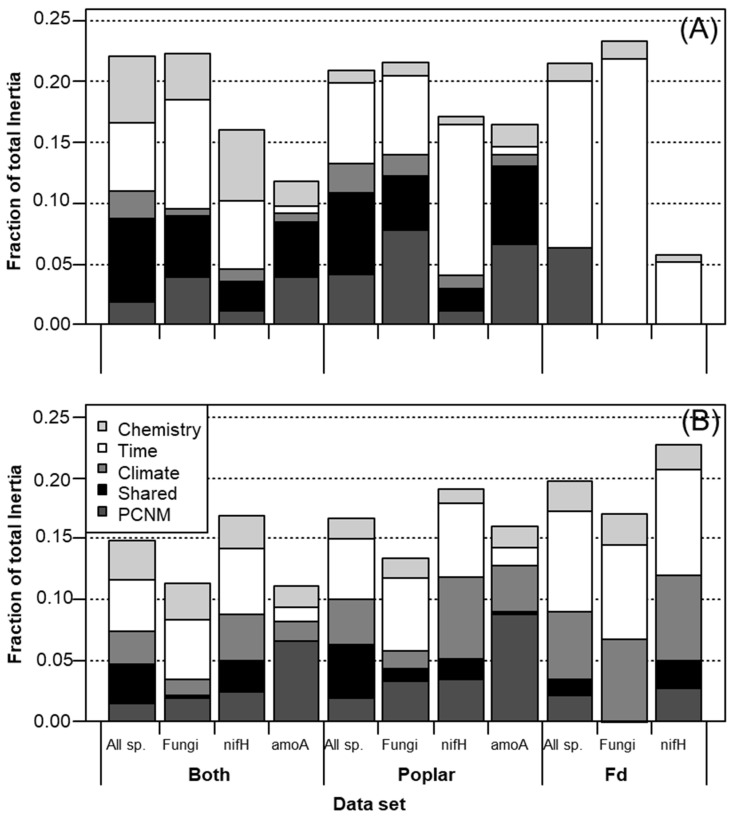
Fraction of microbial community (**A**) structure and (**B**) composition explained by litter chemistry, time, climate, spatial PCNM and shared spatio-climatic effects, using partially constrained ordination. Data used in analyses either included all functional groups (all sp.) and both litter species (both), or subsets of data including only a specific functional group (fungi, *NifH* or *AmoA*) and only poplar or fir litter samples.

Time was significantly associated with up to 9% and 9.1% of the variation in overall microbial community structure and composition, respectively (Table 3—analyses of all taxa for both poplar and fir litter). No temporal effects were detected for the *AmoA* functional group, except for a 1.2% shift in *AmoA* community composition when considering time as a continuous variable (Figure 1A), despite lower explanatory power for overall microbial community structure and composition (4.3% and 5.7%, respectively; Figure 2) compared to categorical analyses. The structure of fungal communities in both poplar and fir litter changed over time (Table 3A and Table 4A). In poplar litter, fungal richness was greatest after 43 months of decay and was lowest during the 7-month sampling period (17 and 14 OTUs on average, respectively), while fungal community evenness generally decreased over time and diversity remained unchanged (*p* = 0.001). In fir litter, fungal richness decreased slightly until 24 months of decay (15–14 OTUs per fir sample, on average, in fir litter; *p* = 0.001), with concurrent increases in evenness and diversity, but was greatest in samples after 43 months (23 OTUs per fir sample, on average; *p* = 0.001).

**Figure 3:**
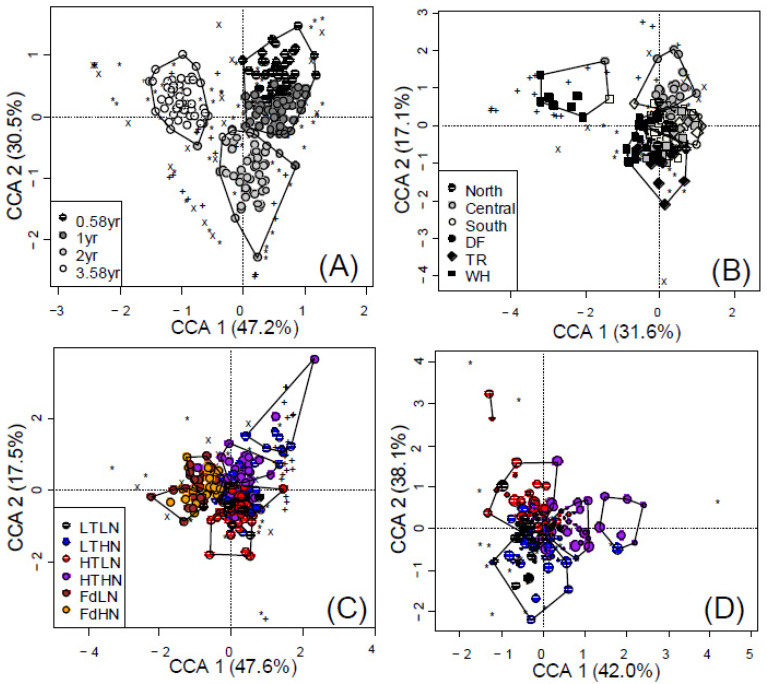
Partially constrained CCA showing the effects of (**A**) time, (**B**) site and (**C**) CT level on microbial composition of fungi (*), *NifH* (+) and *AmoA* (X) communities associated with poplar and Douglas-fir (Fd) leaf litter with low and high nitrogen (LN and HN) and condensed tannin (LT and HT, poplar only) content and after 7, 12, 24 and 43 months of decay. Litter chemistry effects on (**D**) fungi communities associated with poplar litter only, where point size decreases with time. Polygons represent k-means clustering based on CCAs 1 and 2.

The structure of *NifH* communities was only affected by time in poplar litter, not fir (Table 3A and Table 4A). The richness, evenness and diversity of *NifH* communities in poplar litter increased until 24 months of decay, on average being 17.6%, 22.3% and 33.5% greater after 24 months of decay compared to after 7 months of decay (*p* = 0.001). Composition of fungal and *NifH* communities also changed over time, with more pronounced differences found after the first year of decay (Figure 1A and Figure 3A). Overall microbial community composition was significantly associated with 3.1% of changes in the C remaining in litter (*p* = 0.001), which accounted for 1.3% of changes independent of time, litter chemistry, space or climate (*p* = 0.001). Overall community structure was not significantly associated with changes in C remaining in litter (*p* = 0.272), since the majority of associated effects (3.4 of 3.9%) were accounted for by other factors.

### 3.3. Litter Chemistry Effects

Litter type was significantly associated with microbial community structure (5.7%), but generally not when treating poplar and fir litter separately (Table 3A and Table 4A). Fungal richness, evenness and overall diversity were greater in fir than poplar litter (8.3%, 4.0% and 5.9%, respectively). Differences in fungal structure were primarily associated with greater diversity, evenness and, to a lesser extent, richness in litter with lower CT content, while greater AUR was associated with greater richness and less evenness (Table 4A). Greater ash content in fir litter, which was the chemical variable showing the least *Z*-score variance within litter species, was associated with the 18.5% greater *NifH* community evenness and 24.7% lower richness in this litter compared to poplar, as well as with different *NifH* community composition (Table 4A).

When assessing both poplar and fir litter, higher initial N content was associated with greater *AmoA* diversity and different *AmoA* community composition (Table 4A; Figure 1A,C). However, despite not detecting *AmoA* genes in any fir litter samples, N content in poplar litter was only deemed significant in determining *AmoA* community structure and composition when omitting samples collected after 43 months of decay (i.e., only samples decaying for 7, 12 and 24 months; *p* = 0.037 and 0.034, respectively; Table 4).

Overall, litter type was significantly associated with 4.1% of detected variance in microbial community composition (categorical analyses); 3.2% could be attributed to initial N, CT and AUR content in litter (Figure 1C and Figure 2B). Condensed tannin and AUR content accounted for 3% of differences in fungal composition among litter types. All but one of the OTUs were detected poplar samples, while only 60 out of 66 fungal OTUs and 18 out of 23 *NifH* OTUs were detected in fir litter. When treating litter species and functional groups separately, litter chemistry was only significant in describing fungal responses in poplar litter (Table 3B). CT and N treatments were associated with shifts in fungal community composition in poplar litter (Figure 1D), however, this was only significant when omitting samples collected after 43 months of decay (i.e., only samples decaying for 7, 12 and 24 months; *p* = 0.039 and 0.014, respectively; Table 4B). Litter chemistry treatments still accounted for 2.2% and 2.4% of overall changes in fungal OTUs when solely considering poplar (Figure 3D) or fir data sets, respectively (Table 3B); these effects were not distinctly clustered when models included all functional groups and litter species (Figure 3C).

### 3.4. Spatial and Climatic Effects

Site (latitude × zone) accounted for 11.2% and 7.3% of overall microbial community structure and composition, respectively (*p* = 0.001, Figure 3B, Table 3). The structure of each fungal and *NifH* community differed between sites in poplar litter (*p* ≤ 0.022), while only differences in fungal community structure were detected fir litter (*p* = 0.047, Table 3A). All fungal diversity indices were greater at northern sites, especially in the TR and WH zones, while lowest at southern sites. Diversity indices of *NifH* communities in poplar litter were greatest in central sites, especially in the DF zone. Incidence of *AmoA* OTUs was less frequent than for other functional groups, however, community structure was still associated with site in poplar litter (*p* = 0.001, Table 3A). Over time, the 23 *AmoA* OTUs we detected were largely found on poplar litter in NWH and DF sites. Reflecting structural indices, cluster analysis of microbial composition revealed generally different communities in litter decaying at NWH or CDF sites vs. the other remaining sites. Communities in the other sites divided into two groups dominated by northern or southern and central sites (Figure 3B).

Climate variables did not significantly describe microbial community structure in fir litter (*p* > 0.05; Table 4A). Despite accounting for 9.1% of microbial community structure in poplar litter, 6.8% of climatic effects on community structure were shared with spatial effects (Figure 2A), rendering independent climatic effects insignificant (Table 4A). Nonetheless, 3.5% lower MS_min_, which was inversely correlated with PCNM 3 (*r* = −0.514), was associated with 13% greater diversity and 27% greater richness of the fungal community, while 4.9% lower MS, which correlated with PCNM 2 (*r* = 0.619), was associated with 5.8 times greater diversity and 4.6 times greater richness of the *AmoA* community (Table 4A). Increasing TA (1.6 °C) correlated with PCNM 3 (*r* = 0.575) and was associated with lower *NifH* community diversity, richness and evenness (Table 4A). The slightly lower *AmoA* community richness was also associated with greater TA (Table 4A). It was confounded by the joint effects of PCNMs 1, 2 and 3 (Table 4A), although spatio-climatic correlations were low (*r* < 0.316).

Climate accounted for 2.7% of microbial community composition independently, and shared a further 3.2% of effects with PCNM (Figure 2B). Independent climatic effects significantly accounted for community composition for each functional group and litter species subset, except for fungi in poplar litter, where the effects of MS_min_ and MS_max_ were confounded by negative correlations with PCNMs 3 (*r* = −0.514) and 1 (*r* = −0.666), respectively (Table 4B). MS_min_, TA, TS_range_ and PET were all significantly associated with *NifH* community composition in poplar litter, while the effects of TA_min_ were confounded by correlations with PCNM 4 (*r* = 0.752; Table 4B). Nonetheless, TA_min_ was significantly associated with the composition of *AmoA* communities in poplar litter and *NifH* communities in fir litter (Table 4B). TS_range_ was significantly associated with *NifH* community composition in poplar litter and fungal community composition in fir litter (Table 4B). The significant effects of TA_max_ on *AmoA* community composition in poplar (*p* = 0.049) and composition of the fungal community in fir litter (*p* = 0.003) were only detected when sub-setting litter species and functional groups (Table 4B).

**Figure 4:**
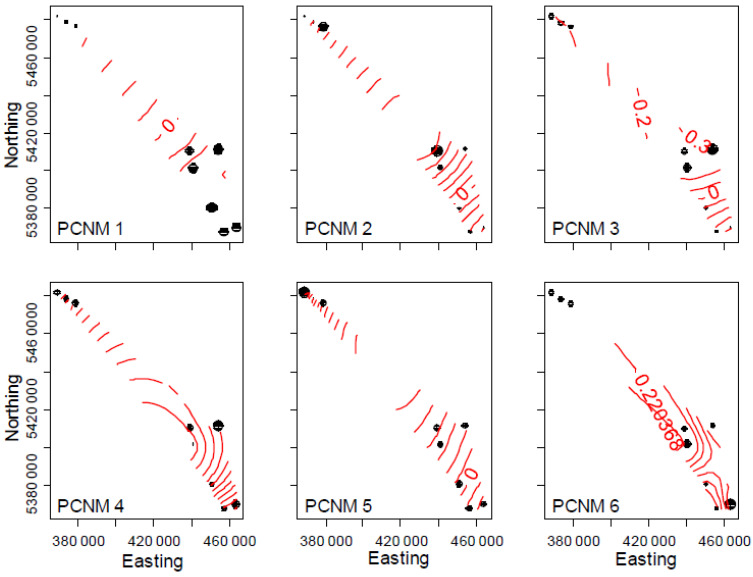
Principal components of neighbor matrices (PCNMs) of spatial layout of field sites. PCNMs 1, 3, 4, 5 and 6 were significantly associated with microbial composition, while PCNMs 1, 2, 3, 4 and 5 were significantly associated with community structure. Point size is proportional to PCNM values for each sampling location.

Spatial PCNM components 1, 3, 4 and 5 were significantly associated with overall microbial community structure (9.4%, *p* = 0.001) while PCNMs 1, 3, 5 and 6 were associated with composition (4.5%, *p* = 0.001; Figure 4). PCNM 3 was the only spatial component associated with microbial composition in fir litter, namely due to correlations with *NifH* community composition in each litter species (*p* < 0.008), but was confounded by climate effects (Table 4B and Figure 2B). Although PCNM 4 was significantly associated with overall microbial community structure in fir litter (*p* = 0.01), most likely due to fungi per joint analyses of poplar and fir samples (Table 4A), no spatial patterns were significantly attributed to the structure of individual functional groups. PCNM 4 was also associated with the composition of *NifH* communities in poplar litter (*p* = 0.045), but correlations with TA_min_ (*r* = 0.752) confounded such effects (Table 4B). In poplar litter, PCNM 1 was associated with the composition of each functional group (*p* < 0.002) and the structure of fungal and *AmoA* communities (*p* = 0.001 and 0.002, respectively).

Despite being correlated with less MS_max_ and greater TA_max_ (*r* = −0.666 and 0.661, respectively), PCNM 1 was only confounded by climate when assessing *NifH* community composition in poplar litter (*p* = 0.139). PCNM 3 was associated with the composition and structure of only fungal and *NifH* communities (*p* < 0.018), but responses were generally confounded by climate. PCNM 2 was uniquely associated with the structure of *AmoA* communities (*p* = 0.014), although confounded by correlations with MS (*r* = 0.619), while PCNM 5 was associated with both structure and composition (*p* = 0.001). PCNM 5 was also significant in describing overall *NifH* community composition (*p* = 0.039), but not when poplar and fir data were treated separately nor when accounting for climate. PCNM 6 was only associated with fungal community composition in poplar litter (*p* = 0.017).

## 4. Discussion

Litter chemistry and weather affected the composition of fungi and N-cycling communities in decaying litter, often reflecting trends in C decay and nutrient cycling. Spatial layout and temporal effects, which could not be completely isolated from climate effects, further explained significant portions of the structure and composition of microbial communities. Analyzing subsets of microbial communities in decaying leaf litter sometimes revealed more subtle (or incongruent) effects that were masked when all community data were used (e.g., the effects of TA_max_ on *AmoA* and fungal community structure), similar to observations made of baseline soil communities [40]. Carefully designed cluster analyses of constrained ordinations, accompanied by independent testing of explanatory variables (within potentially nested effects), revealed more than would otherwise be apparent (e.g., Figure 3C,D). We were able to observe macroecological trends impacting soil microbes, such as CT and N effects nested within time and litter species.

### 4.1. Ammonifying Communities Are Altered by Weather and Litter Nitrogen

Temporal shifts in the composition of the *AmoA* community progressed gradually over time, and hence could not be detected for this rarer functional group using only pairwise-like comparisons (Table 3). The increasing detection of *AmoA* functional genes in decaying litter with high N and at warmer sites with ample soil moisture is indicative of increasing ammonia availability in such rapidly decaying litter, albeit at earlier and later time frames, respectively [19]. Similarly, warmer temperatures have been associated with the biogeography of *AmoA* community composition in the forest floors at our sites [63], indicating that the influence of climate on *AmoA* communities in decaying litter is reflective of long-term trends at our sites. The absence of *AmoA* genes in fir litter decaying side-by-side with poplar litter (where they were detected) confirms that site-specific inoculation is not the reason for observed effects, nor is the endemicity of the litter.

Increased air temperature may alter the N forms available to trees, favoring species with affinities for NO_3_^−^, unless the annual additive short-term effects of reduced litter N can offset the longer-term consequences of temperature. In the presence of a favorable future precipitation and humidity regime, changes in nutrient status in warmer climates could favor the growth of western hemlock, which preferentially takes up NO_3_^−^ in contrast to fir [64,65]. This would be inconsistent with a hypothesized westward shift of the transition zone between coastal western hemlock (CWH) and coastal Douglas-fir (CDF) BGC zones [66].

The rapid decay rate of fir litter one year after the onset of decomposition [19] and concurrent increasing N mineralization in this litter type (unpublished) were never accompanied by the emergence of *AmoA* populations (i.e., never detected). We expect that mycorrhizae translocated surplus N from the in situ fir litter type to nearby trees or plants, preventing ammonia accumulation at the site of decay.

### 4.2. Weather Can Account for Early Succession in Fungal Composition

The chemical, climatic and PCNM variables selected in our final models thoroughly described the range of effects in our experimental design, since less than 1% of the effects of litter chemistry or site were not accounted for by continuous variables describing such treatments. The temporal effects on microbial community structure and composition not accounted for when using continuous time variables (4.7% and 3.4%, respectively) indicated non-linear trends in fungal and *NifH* community succession during decay. Successional shifts in fungal community composition were more pronounced after 24 months, and especially after 43 months of decay (Figure 3A), and did not affect fungal diversity in our decaying poplar and fir litter, similar to rapid successional changes observed in oak systems [41]. Increasing time periods between successive sampling could be responsible for the increasing divergence of community composition between sampling periods, and potentially indicate distinct annual shifts in community composition.

In a separate analysis, we were able to attribute a significant portion of the succession in fungal communities over the first 24 months of decay to weather regimes, when adjusting temperature and moisture variables over time in order to only reflect the weather litter bags were exposed to (see Supplementary Material for details). The ability of weather to explain successional changes in microbial community structure and composition runs counter to the classical view of microbial succession as a response to changing resource availability. Rapid turnover of microbial communities has been observed in response to climatic stress [67]. Our observations of microbial succession as a response to ongoing weather variation would support such findings, although confounded by exposure time and starting inoculum. Since climate impacts microbial biogeography [40], it is unclear if weather is directly accountable for observed fungal succession, or if instead climate is indirectly affecting succession via selection of available microbial inoculum in soils. Lin et al. [68] studied disturbance and fresh litter deposition in the forests of another conifer, *Cryptomeria japonica*, and showed that divergent fungal communities in different plots essentially converged and became identical after 21 months. Given the 43-month duration of our experiment, we therefore expect that the differences we observed in our communities were ultimately related to the impact of climate on community succession in the litter bags and/or its impact on the composition of the parent community. Experimental protocols using high-throughput sequencing for identifying microbial communities would allow direct comparison of communities in soils and decaying litter. Our results warrant further investigations into the extent of climate-induced microbial succession.

### 4.3. Short-Term Effects on Fungal Communities Driven by Litter Chemistry

Climate-induced shifts in foliar CT and N content [11,12,13,14] would affect litter fall chemistry and thus could alter below-ground fungal communities. The ranges of CT content (76–278 mg g^−1^) and N content (7.5–15 mg g^−1^) within poplar litter affected fungal composition up until 24 months of decay, and yet were not associated with the structure of fungal communities. These observations contradict our predictions that CTs inhibit overall microbial diversity. In this case, higher CT levels were simply associated with different fungal communities; we presume that a different cohort of decay fungi was selected from the more diverse background community. The effects of litter chemistry on community structure were mostly driven by litter species; parameters distinguishing poplar from fir litter (e.g., CT form and cutin concentration) were more important than intra-specific variation in CT and N concentrations in determining community diversity indexes. Intra-specific variation in CT and N concentrations may not be broad enough to elucidate relationships between litter chemistry and microbial community structure (i.e., diversity, richness and evenness) or alternatively the cohorts of decay fungi available to decompose litter are diverse enough that CT levels do not matter.

The changes in fungal composition associated with greater CT and lower N content were accompanied by reduced rates of decay [19]. The resolution of PCR-DGGE allowed linkage of changes in microbial community composition to C loss. The lack of association between our diversity indices (i.e., community structure) and litter-decay status supports previous findings using different microbial screening tools [69].

The different microbial communities detected on decaying poplar and fir litter mirror their differing rates of decay [19] and are likely indicative of distinct nutrient flows. It is possible that some of the fungal OTUs affected by our CT treatments were mycorrhizal species, as was the case in a study of the effects of CT from black spruce and sheep laurel [70]. Mycorrhizae strongly influence competition for nutrients between plants and decomposers, as well as rates of C sequestration [71]. Feedback loops between above- and below-ground communities were also demonstrated in grass/shrub systems, where plant community traits (e.g., C/N ratios) reflect their fundamental niche and generate positive feedback loops via microbial selection that can perpetuate the nutrient status of an ecosystem [72].

### 4.4. Diazotrophs Differ among Litter Types

Litter species had a strong influence on *NifH* communities and how they were affected by climate. Different *NifH* OTUs colonized decaying litter from poplar and fir, hence some of the detected diazotroph species may be species- or litter-specific. Winder et al. [37] identified several diazotrophic endophytes of poplar during microcosm incubations of poplar litter, a reminder that diazotrophs in our litter bags may not have been limited to free-living species. *NifH* community composition was further affected by the annual range in soil temperature in poplar litter, and by the minimum annual air temperature in fir litter (Table 4B). Apart from being a potential source of essential mineral elements (e.g., Mg, Fe, Mo), it is unclear how slight differences in initial ash content could be responsible for differing *NifH* community structure or composition between poplar and fir litter. Since ash was correlated with CT differences between litter species (*r* = −0.763), it is possible that the amount or type of CT, even in low-CT poplar treatments, may have selected for different diazotrophic species. Alternatively, ash content may correlate with other compounds or metabolites that we did not measure and vary in content between fir and poplar litters (e.g., waxes and cutins). Selective colonization may also be simply the result of significantly different C-decay trajectories and nutrient availability between litter species during decay, as suggested by Widmer et al. [54]. We generally detected more *NifH* genes (i.e., more richness) in poplar litter compared to fir litter. The slower decay rates for poplar litter from 7 to 43 months of decay compared to fir litter could suggest low N availability in this litter during this time period, however, the presence of genes from specific microbial communities (such as N-cycling soil bacteria) may not necessarily directly reflect ecological activity [73].

## 5. Conclusions

High CT and low N concentrations in leaf litter were associated with changes in microbial community composition, especially fungi, which may be directly responsible for slower early (<2 years) decay rates of poplar litter and lead to a net annual sequestration of litter carbon in forest soils. Contrary to our hypothesis, high CT and low N treatments did not inhibit microbial colonization or diversity. The effects of initial litter chemistry, AUR (35.3–41.5% dw), CT (2.1–29.1% dw) and N (0.61–1.57% dw) on microbial community composition in decaying litter were less than the joint effects of 1.6 and 3.4 °C differences in annual mean and minimum air temperature, respectively, and 4.1% and 26.9% differences in annual minimum and maximum soil moisture, respectively (Figure 2). Long-term microbial composition in forest soils may therefore be primarily affected by future climates, especially since the effects of litter chemistry diminish over time as the compositions of different types of litter converge. Furthermore, shared spatio-climatic and temporal–climatic effects made up a considerable portion of climate effects, and warrant further investigation in the face of climate change. The ability of available microbial inoculum to colonize both in situ and ex situ litter types shows promise for the capacity of below-ground systems to adapt to large-scale migration (natural or assisted) of trees in response to climate. However, differences in community assemblages were indicative of functional shifts that could compound over time and alter decay rates as climate change progresses. These differences should be carefully monitored where regions experience significant natural or deliberate shifts in forest cover. Baseline assessments of microbial biogeography in soils were helpful in interpreting our experimental results and will be of increasing importance in the analysis of future impacts of climate change on such a critical and lesser-known part of our ecosystems.

## Figures and Tables

**Table 1:** Initial litter chemistry of *Pseudotsuga menziesii* (fir = Fd) and *Populus angustifolia* (poplar) leaf litter with low and high nitrogen (LN and HN) and condensed tannins (LT and HT, poplar only), including carbon (C), nitrogen (N) and their ratio (C/N), phenols, total condensed tannins (CT) and proximate chemistry. Mean values for each litter type were used for modeling microbial community responses during decay. Values (±SE) are based on dry weight (70 °C overnight) and calculated from 6 and 4 replicates (for poplar and Fd litter, respectively) or 3 replicates (for proximate chemical fractions).

Species	Litter Type	C (mg g^−1^)	N (mg g^−1^)	C/N	Phenols (mg g^−1^)	CT (mg g^−1^)
*Populus angustifolia*	LT LN	457.0 ± 1.1	8.1 ± 0.2	56.39 ±1.45	18.3 ± 3.4	66.4 ± 8.1
	LT HN	461.4 ± 0.7	15.7 ± 0.3	29.42 ±0.55	19.2 ± 4.4	85.8 ± 6.4
	HT LN	481.8 ± 1.2	6.9 ± 0.2	70.36 ±1.72	60.8 ± 6.8	264.9 ± 7.9
	HT HN	477.5 ± 1.3	14.3 ± 0.1	33.32 ±0.40	54.3 ± 4.8	291.5 ± 5.3
*Pseudotsuga menziesii*	Fd LN	512.6 ± 1.5	6.1 ± 0.1	83.52 ±1.37	19.0 ± 3.1	20.8 ± 0.4
	Fd HN	511.0 ± 0.5	11.2 ± 0.2	45.46 ±0.76	18.4 ± 1.5	20.9 ±0.3
**Species**	**Litter Type**	**NPE ^a^** **(mg g^−1^)**	**WSE ^b^** **(mg g^−1^)**	**AHF ^c^** **(mg g^−1^)**	**AUR ^d^** **(mg g^−1^)**	**Ash** **(mg g^−1^)**
*Populus angustifolia*	LT LN	137.6 ± 15.2	130.7 ± 0.1	304.2 ± 5.0	414.8 ± 6.6	12.7 ± 0.6
	LT HN	152.4 ± 5.6	128.4 ± 7.5	307.8 ± 4.4	398.1 ± 2.7	13.3 ± 1.6
	HT LN	177.7 ± 15.2	154.0 ± 11.1	283.8 ± 10.9	373.1 ± 5.4	11.4 ± 0.8
	HT HN	230.2 ± 6.5	132.3 ± 12.4	274.3 ± 3.0	353.0 ± 5.5	10.1 ± 0.5
*Pseudotsuga menziesii*	Fd LN	99.5 ± 3.5	119.5 ± 1.0	362.6 ± 0.6	394.0 ± 3.4	24.4 ± 0.6
	Fd HN	121.6 ± 3.2	117.2 ± 1.5	355.1 ± 1.2	382.6 ± 0.5	23.4 ± 0.4

^a^ Non-polar extractables, ^b^ water-soluble extractables, ^c^ acid-hydrolyzable forage, ^d^ acid-unhydrolyzable residue.

**Table 2:** In situ climatic conditions measured at nine sites spanning three latitudes (north, central, south) and two biogeoclimatic (BGC) zones (coastal western hemlock (WH), coastal Douglas-fir (DF) or transition between the two (TR)) over 48 months (1 May 2011 to 30 April 2015).

Latitude	BEC Zone	Soil Moisture (%) ^a^	Soil Temp. (°C) ^a^	Air Temp. (°C) ^b^	PET ^c^
**North**	DF	13.93 [3.85, 40.97]	9.55 [3.73, 15.37]	8.99 [−4.86, 23.90]	88.2
	TR	12.97 [2.77, 45.56]	9.64 [2.84, 15.85]	9.60 [−4.30, 27.18]	92.5
	WH	9.05 [3.03, 20.62]	8.97 [2.96, 14.71]	8.97 [−5.90, 26.32]	90.5
**Central**	DF	9.83 [2.85, 23.07]	9.87 [3.37, 16.92]	10.09 [−3.52, 26.62]	93.6
	TR	9.2 [1.92, 19.52]	9.68 [2.47, 17.09]	9.44 [−6.67, 29.03]	92.7
	WH	13.86 [3.50, 26.84]	8.58 [2.14, 15.29]	8.72 [−5.56, 31.26]	90.3
**South**	DF	11.93 [4.03, 24.72]	9.94 [3.91, 16.18]	9.59 [−5.18, 30.00]	89.3
	TR	13.1 [3.47, 25.85]	8.96 [2.76, 14.48]	8.44 [−6.97, 28.16]	87.5
	WH	11.25 [6.00, 18.68]	8.40 [2.31, 13.55]	8.61 [−5.97, 28.50]	87.8

^a^ Mean annual [mean of annual minimums, maximums] soil moisture or temperature measured 30 cm below forest floor (abbreviated as MS, MS_min_ and MS_max_ for soil moisture and TS, TS_min_ and TS_max_ for soil temperature). ^b^ Mean annual [mean of annual minimums, maximums] air temperature measured 30 cm above forest floor (abbreviated TA, TAmin and TAmax). ^c^ Mean potential evapotranspiration from May to September, using the Thornthwaite method.

**Table 3:** Significance (*p*-values) of categorical models and predictors in predicting microbial community structure (**A**; Shannon’s diversity, richness and Pielou’s evenness) or composition (**B**; presence/absence). Models constrained by categorical time, litter type and site predictors (constrained by treatment) or by the DNA concentration and λ absorbance (constrained by DNA) were reduced until only significant predictors remained (α-level = 0.05), with non-significant variables labelled ‘ns’. Best fit categorical models were then assessed after removing the effects of DNA quality on microbial responses (partially constrained by DNA), with ‘NA’ labeling cases where DNA quality is not significant. Data from poplar and Douglas-fir litter were treated separately (poplar only and fir only, respectively) and jointly (poplar and fir). Fungi, *AmoA* and *NifH* functional groups were also treated separately and jointly (all taxa).

(A) Structure		Poplar and Fir				Poplar Only				Fir Only ^a^		
		All taxa	Fungi	*NifH*	*AmoA*	All taxa	Fungi	*NifH*	*AmoA*	All taxa	Fungi	*NifH*
**Constrained by treatments**	time	<0.001	<0.001	<0.001	ns	<0.001	<0.001	<0.001	ns	<0.001	<0.001	ns
	litter type	<0.001	0.003	0.005	ns	ns	ns	ns	ns	ns	ns	ns
	site	<0.001	<0.001	0.008	0.003	<0.001	<0.001	0.031	<0.001	0.013	0.026	ns
	model	<0.001	<0.001	<0.001	<0.001	<0.001	<0.001	0.001	<0.001	<0.001	<0.001	ns
**Constrained by DNA**	concentration	ns	ns	ns	0.02	0.027	ns	ns	0.008	0.043	ns	0.018
	260/280 ^b^	0.003	0.033	0.005	ns	ns	ns	0.036	ns	ns	ns	ns
	260/230 ^b^	ns	ns	ns	ns	ns	ns	ns	ns	0.026	0.002	ns
	model	0.002	0.029	0.005	0.023	0.021	ns	0.035	0.009	0.016	0.002	0.018
**Partially constrained by DNA**	time	<0.001	<0.001	<0.001	ns	<0.001	NA	0.001	ns	<0.001	<0.001	ns
	litter type	0.003	0.034	0.048	ns	ns	NA	ns	ns	ns	ns	ns
	site	<0.001	<0.001	0.008	<0.001	<0.001	NA	0.022	<0.001	0.068	0.047	ns
	model	<0.001	<0.001	<0.001	<0.001	<0.001	NA	0.001	<0.001	<0.001	<0.001	ns
**(B) Composition**		**Poplar and Fir**				**Poplar Only**				**Fir Only ^a^**		
		**All taxa**	**Fungi**	***NifH***	***AmoA***	**All taxa**	**Fungi**	***NifH***	***AmoA***	**All taxa**	**Fungi**	***NifH***
**Constrained by treatments**	time	0.005	<0.001	<0.001	ns	<0.001	<0.001	<0.001	ns	<0.001	<0.001	<0.001
	litter type	0.005	<0.001	<0.001	ns	0.037	0.035	ns	ns	0.04	0.032	ns
	site	0.005	<0.001	<0.001	<0.001	<0.001	<0.001	<0.001	<0.001	<0.001	<0.001	0.002
	model	<0.001	<0.001	<0.001	<0.001	<0.001	<0.001	<0.001	<0.001	<0.001	<0.001	<0.001
**Constrained by DNA**	concentration	0.005	<0.001	0.034	0.009	0.002	0.008	ns	<0.001	ns	0.029	ns
	260/280 ^b^	0.007	0.009	<0.001	ns	ns	ns	0.004	ns	ns	ns	<0.007
	260/230 ^b^	ns	ns	ns	ns	0.03	ns	0.007	ns	ns	ns	ns
	model	<0.001	<0.001	<0.001	0.006	0.012	0.006	<0.001	<0.001	ns	0.043	0.008
**Partially constrained by DNA**	time	<0.001	<0.001	<0.001	ns	<0.001	<0.001	<0.001	ns	NA	<0.001	<0.001
	litter type	<0.001	<0.001	<0.001	ns	0.045	0.041	ns	ns	NA	0.076	ns
	site	<0.001	<0.001	<0.001	<0.001	<0.001	<0.001	<0.001	<0.001	NA	<0.001	<0.001
	model	<0.001	<0.001	<0.001	<0.001	<0.001	<0.001	<0.001	<0.001	NA	<0.001	<0.001

^a^*AmoA* genes were not detected in fir litter, therefore the column was omitted. ^b^ Wavelength absorption ratios of extracted DNA solutions.

**Table 4:** Significance (*p*-values) of continuous models and predictors in predicting microbial community structure (**A**; Shannon’s diversity, richness and Pielou’s evenness) or composition (**B**; presence/absence). Partially constrained ordination analyses were used to test the effects of continuous time, initial litter chemistry, climate or principal coordinates of neighbor matrix (PCNM) predictors, while removing other respective effects. Climate and PCNM variables (and litter chemistry in the case of joint poplar and Fd analyses) were first reduced until only significant predictors remained (α-level = 0.05). Cases where no variables were deemed significant and when all of the variance accorded to a variable is confounded by partial constraints are labeled by ‘ns’. Data from poplar and Douglas-fir litter were treated separately (poplar only and fir only, respectively) and jointly (poplar and fir). Fungi, *AmoA* and *NifH* functional groups were also treated separately and jointly (all taxa). The column for *AmoA* is not included when using only Fd data since this gene was never detected in this litter type. Mean annual range in soil temperature is designated ‘TS_range_’. Refer to Table 1 and Table 2 for other abbreviation definitions.

(A) Structure		Poplar and Fir				Poplar Only				Fir Only ^a^		
		All taxa	Fungi	*NifH*	*AmoA*	All taxa	Fungi	*NifH*	*AmoA*	All taxa	Fungi	*NifH*
**Partially constrained model significance**	time	<0.001	<0.001	<0.001	0.366	<0.001	<0.001	<0.001	0.346	<0.001	<0.001	0.1
	litter chem.	<0.001	0.004	<0.001	0.004	0.508	0.376	0.611	0.183	0.461	0.451	0.734
	climate	0.192	0.546	0.121	0.631	0.188	0.064	0.162	0.614	ns	ns	ns
	PCNM	0.12	0.011	0.076	0.008	0.012	<0.001	0.157	0.002	0.012	ns	ns
**Predictor significance**	litter chem.	N (0.026) CT (0.008) AUR (<0.001)	CT (0.027) AUR (0.029)	Ash (<0.001)	N (0.007)	CT (0.564) N (0.297)	CT (0.173) N (0.85)	CT (0.766) N (0.341)	CT (0.711) N ^b^ (0.078)	N (0.484)	N (0.424)	N (0.717)
	climate	MS (0.068) MS_min_ (0.075) MS_max_ (0.72) TA (0.735) TA_min_ (ns)	MS_min_ (0.239) TA_min_ (0.959)	TA (0.121)	MS (0.771) TA (0.378)	MS (0.131) MS_max_ (0.433) TA (0.3)	MS_min_ (0.071)	TA (0.195)	MS (0.796) TA (0.332)	ns	ns	ns
	PCNM	PCNM 1 (0.007) PCNM 3 (0.826) PCNM 4 (0.682) PCNM 5 (ns)	PCNM 1 (0.003) PCNM 3 (0.672) PCNM 4 (0.462)	PCNM 3 (0.078)	PCNM 1 (0.003) PCNM 2 (0.583) PCNM 5 (0.122)	PCNM 1 (<0.001) PCNM 3 (0.219) PCNM 5 (0.832)	PCNM 1 (0.001) PCNM 3 (0.429)	PCNM 3 (0.151)	PCNM 1 (0.002) PCNM 2 (0.512) PCNM 5 (0.044)	PCNM 4 (0.01)	ns	ns
**(B) Composition**		**Poplar and Fir**				**Poplar Only**				**Fir Only ^a^**		
		**All taxa**	**Fungi**	***NifH***	***AmoA***	**All taxa**	**Fungi**	***NifH***	***AmoA***	**All taxa**	**Fungi**	***NifH***
**Model significance**	time	<0.001	<0.001	<0.001	0.033	<0.001	<0.001	<0.001	0.031	<0.001	<0.001	<0.001
	litter chem.	<0.001	<0.001	<0.001	0.002	0.023	0.051	0.718	0.228	0.08	0.074	0.268
	climate	<0.001	0.03	<0.001	0.008	<0.001	0.153	<0.001	0.002	0.003	<0.001	0.003
	PCNM	0.004	<0.001	<0.001	<0.001	0.002	<0.001	0.002	<0.001	0.296	ns	0.131
**Predictor significance**	litter chem.	N (<0.001) CT (<0.001) AUR (<0.001)	CT (0.003) AUR (<0.001) Ash (<0.001)	Ash (<0.001)	N (0.002)	CT ^d^ (0.162) N (0.024)	CT ^b,c^ (0.059) N ^b^ (0.141)	CT (0.577) N (0.692)	CT (0.533) N ^b^ (0.132)	N (0.076)	N (0.077)	N (0.271)
	climate	MS_min_ (0.009) MS_max_ (0.005) TA (0.03) TA_min_ (0.683) TS_range_^d^ (ns) PET (ns)	MS_min_ (0.012) TS_range_ (0.334)	MS_min_ (0.034) TA_min_ (0.026) TS_min_ (<0.001) TS_range_ (0.016) PET (ns)	TA_min_ (0.013)	MS_min_ (0.016) MS_max_ (0.006) TA (0.014) TA_min_ (0.628) TS_range_ (ns) PET (ns)	MS_min_ (0.474) MS_max_ (0.078)	MS_min_ (0.005) TA (0.01) TA_min_ (0.058) TS_range_ (0.001) PET (0.006)	TA_min_ (0.001) TA_max_ (0.049)	TA_min_ (0.076) TS_range_ (0.005)	TA_max_ (0.003) TS_range_ (0.01)	MS (0.095) TA_min_ (0.002)
	PCNM	PCNM 1 (0.002) PCNM 3 (0.215) PCNM 5 (ns) PCNM 6 (ns)	PCNM 1 (<0.001) PCNM 6 (0.01)	PCNM 1 (0.023) PCNM 3 (0.002) PCNM 4 (0.093) PCNM 5 (ns)	PCNM 1 (<0.001) PCNM 5 (0.001)	PCNM 1 (0.004) PCNM 3 (0.074) PCNM 5 (ns) PCNM 6 (ns)	PCNM 1 (<0.001) PCNM 3 (0.251) PCNM 6 (0.007)	PCNM 1 (0.139) PCNM 3 (0.001) PCNM 4 (0.078)	PCNM 1 (0.002) PCNM 5 (<0.001)	PCNM 3 (0.304)	ns	PCNM 3 (0.131)

^a^*AmoA* genes were not detected in fir litter, therefore the column was omitted. ^b^ Significant when only considering litter decaying for 7, 12 and 24 months. ^c^ Significant when using categorical time predictors. ^d^ Significant when using categorical time predictors while only considering litter decaying for 7, 12 and 24 months.

## Data Availability

Raw data are available on request to the lead author.

## Supplementary Materials

The following supporting information can be downloaded at: https://www.mdpi.com/article/10.3390/jof8070735/s1, Supplementary analysis of fungal communities associated with decay of Douglas-fir and poplar leaf litter over 24 months. Experimental design, analyses and model selection were performed as for data covering 43 months of data, however, continuous climate variables at each site were allowed to change over time in order to only reflect the time period litter was exposed to in the field, spatial effects (PCNM) were included in categorical modeling and additional predictors were included in modeling. Figure S1: Principal coordinates of neighbor matrices (PCNMs) of field-site geographic locations used in modeling. PCNMs 1, 3, 4 and 6 were associated with fungal community structure while only PCNMs 1 and 3 with composition; Figure S2: Fraction of (A) fungal community structure (diversity, richness, evenness) or (B) fungal community composition (OTU pres./abs.) explained by categorical or continuous predictors representing space, year, climate, litter chemistry or shared effects (shared), using RDA or CCA, respectively. Asterisks mark when conditioned models used to calculate a given fraction are significant (permutation test for RDA or CCA *p* < 0.05); Figure S3: CCA of fungal community composition from decaying leaf litter samples in response to litter type (categorical constraints) omitting the effects of years of decay, latitude, zone and space; Figure S4: CCA of fungal composition in response to: (A) litter chemistry alone (ratio of insoluble CT to N (insol.N), C/N ratio (C.N) and % C (C); 3.7% of total variance), (B) climate alone (maximum hourly and minimum mean daily soil moisture (MS_max_, dMS_min_), mean daily and minimum mean daily air temperature (dTA, dTA_min_), and frost-free days (FFDs); 7.2% of total variance) and (C) both chemistry and climate (11% of total variance).
