# Supplementary material for: Fungal Community Composition as Affected by Litter Chemistry and Weather during Four Years of Litter Decomposition in Rainshadow Coastal Douglas-Fir Forests
Source: J Fungi (Basel). 2022 Jul 16;8(7):735. doi: 10.3390/jof8070735 (PMC9323820; doi:10.3390/jof8070735)
Supplement: Supplementary file 1 [file jof-08-00735-s001.zip › jof-1758396-supplementary.pdf]

Supplementary Material
Journal of Fungi-1758396

Shay, Winder, Constabel and Trofymow.

Fungal community composition as affected by litter chemistry and weather during four years of litter decomposition in rainshadow coastal Douglas-fir forests.

Supplementary analysis of fungal communities associated with decay of Douglas-fir and poplar leaf litter over 24 months. Experimental design, analyses and model selection were performed as for data covering 43 months of data, however continuous climate variables at each site were allowed to change over time in order to only reflect the time period litter was exposed to in the field, spatial effects (PCNM) were included in categorical modelling, and additional predictors were included in modelling.

Results

Spatial PCNM components 1, 3, 4 and 6 were associated with fungal community structure while only PCNM 1 and 3 were associated with composition (Figure S1). Although latitude was associated with variability in structure and composition, concurrent correlations with PCNM1 and 3 (r = 0.8863 and -0.414, respectively; $p < 0.05$) confounded significance, even when only considering poplar samples. PCNM 4 and 6 correlate most strongly with zone (r = 0.591 and -0.235, respectively; $p < 0.05$).

Fungal community structure in decaying leaf litter was not affected by years of decay or zone (Figure S2A). Temperature gradients accounted for 5.8% of community structure, independent of geographical space (Figure S2A). Diversity and richness increased with greater total potential evapotranspiration, while evenness decreased with increasing number of frost free days. A small fraction (2.5%) of differences in fungal community structure can be attributed to greater evenness (3% on average) on fir than poplar litter (Figure S2A).

Fungal community composition was most associated with litter chemistry (3.7-4.9%) and climate (2.9-7.2%) followed by years of decay (0.7-3.7%) and spatial distance (1.3-1.7%) (Figure S2B). Most OTUs (82.4%) did not correlate with any measured factors; for some, but not all, due to their ubiquity. Some (1.2%) of the effects of litter type on community composition (Figure S3) were not accounted for by continuous chemical factors (Figure S4A), suggesting that additional chemical components influence fungal colonization of decaying litter.

The effects of initial litter chemistry, C (461-513 mg g^{-1} dw), insoluble CT (11-125 mg g^{-1} dw) and N (6.1-15.7 mg g^{-1} dw) on fungal composition in decaying litter (Figure S4A) were less than joint effects of 6.3 and 10.6°C increases in mean and max air temperature, respectively, and 9.2 and 32.2% decreases in min and max soil moisture, respectively (Figure S4B, Figure S2B). Furthermore, the majority of temporal effects can be accounted for by changes in weather exposure, indicating that climate can account for trends in fungal succession in decaying litter.

Community composition of decay-associated fungi may be altered by ~11% as a result of climate change (Figure S4C), with potential implications on C sequestration and nutrient cycling.

Figures for analysis of fungal communities over 24 months of litter decay

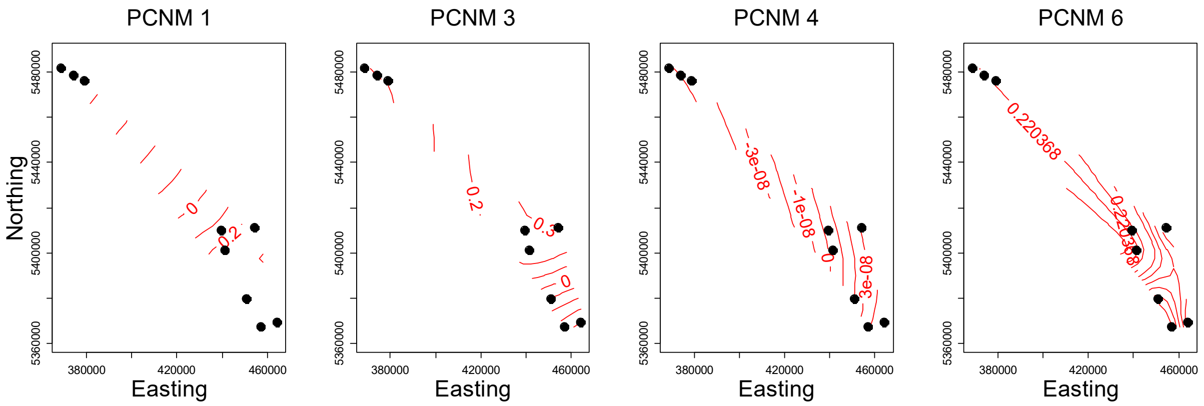

Figure S1. Principal coordinates of neighbor matrices (PCNM) of field-site geographic locations used in modelling. PCNM 1, 3, 4 and 6 were associated with fungal community structure while only PCNM 1 and 3 with composition. Although latitude was associated with structure and composition, concurrent correlations with PCNM 1 and 3 (r = 0.8863 and -0.4138 respectively, p < 0.05) confounded significance, even when only considering poplar samples. PCNM 4 and 6 correlate with zone (r = -0.5915 and -0.2347 respectively, p < 0.05).

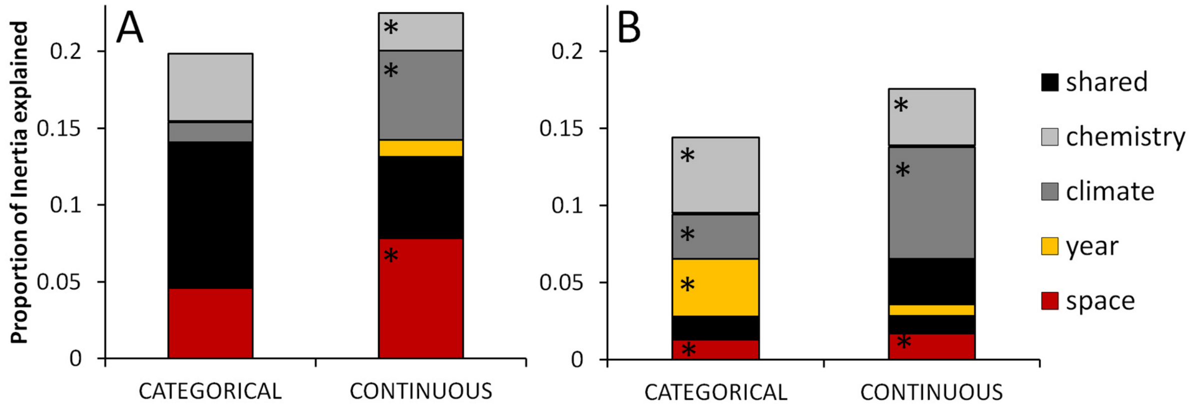

Figure S2. Fraction of (A) fungal community structure (diversity, richness, evenness) or (B) fungal community composition (OTU pres./abs.) explained by categorical or continuous predictors representing space, year, climate, litter chemistry or shared effects (shared), using RDA or CCA respectively. Asterisks mark when conditioned models used to calculate a given fraction are significant (Permutation test for RDA or CCA p < 0.05).

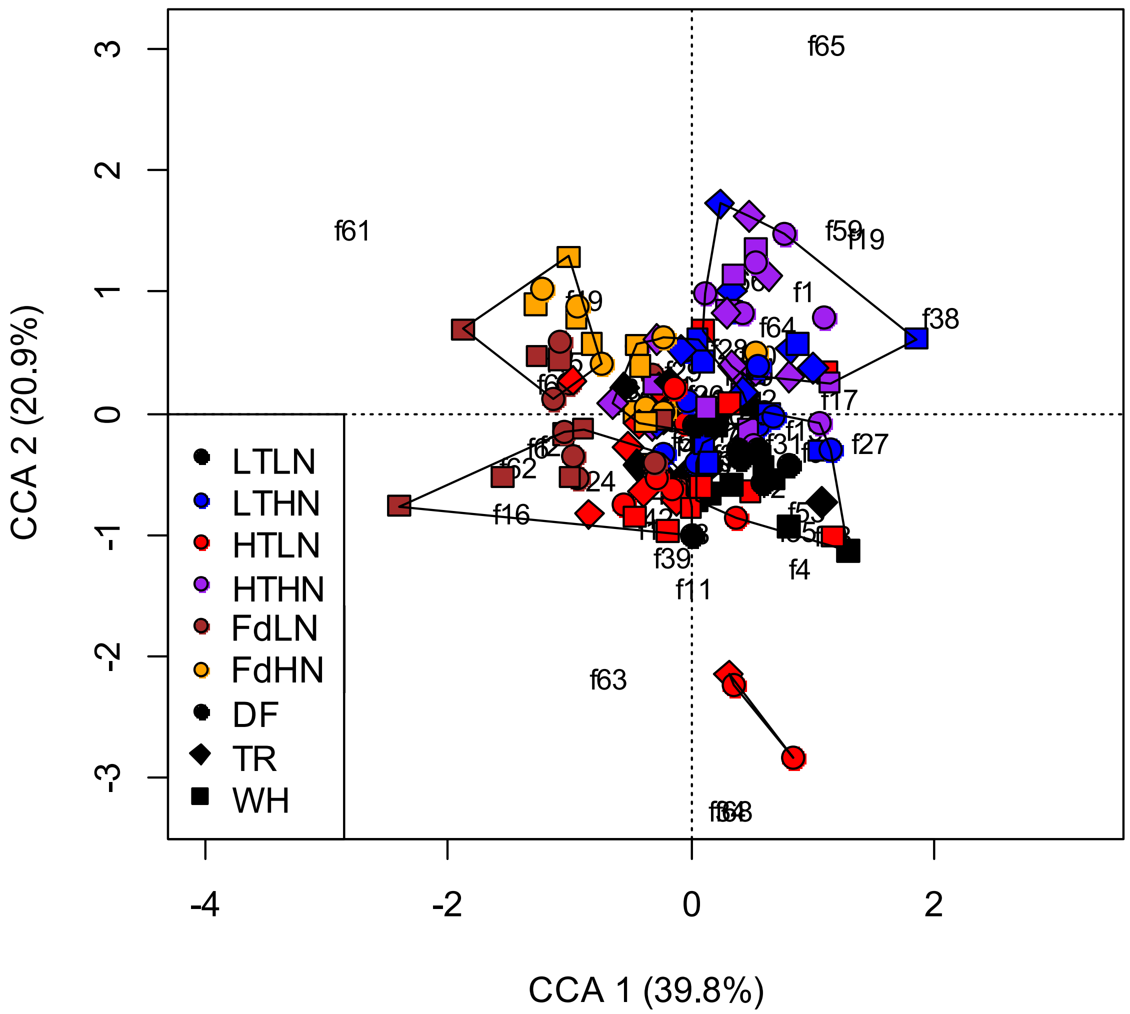

Figure S3. CCA of fungal community composition from decaying leaf litter samples in response to litter type (categorical constraints) omitting the effects of years of decay, latitude, zone and space. Litter type accounted for 4.88% of fungal community composition (3.22% when only considering poplar litter). Polygons represent k-means clustering using CCA components 1 and 2. Each f1 to f68 represents a unique OTU. All responses were scaled to unit variance. Percent inertia explained by each CCA component is in parenthesis next to axis label.

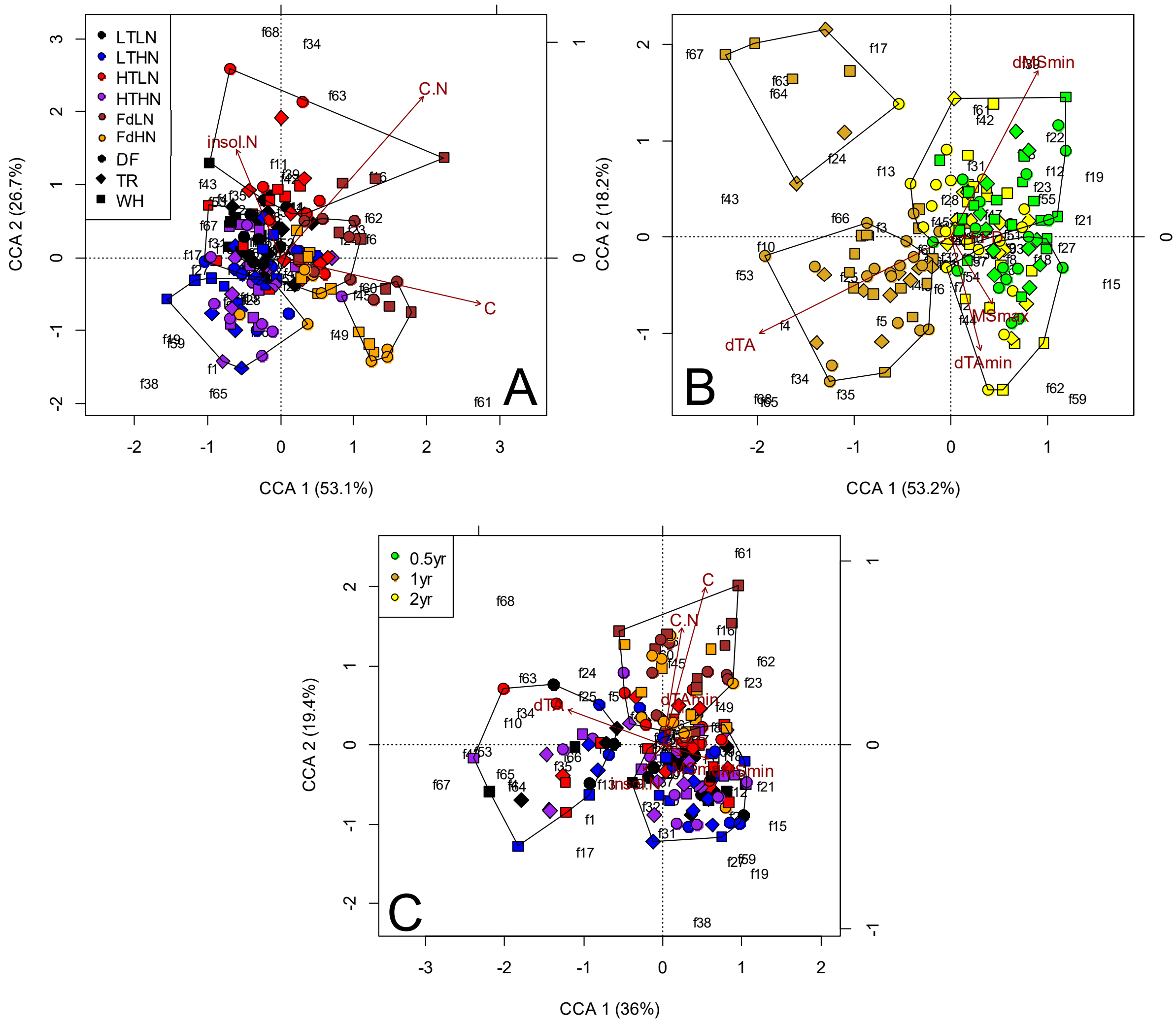

Figure S4. CCA of fungal composition in response to: (A) litter chemistry alone [ratio of insoluble CT to N (insol.N), C/N ratio (C.N) and % C (C); 3.7% of total variance], (B) climate alone [maximum hourly and minimum mean daily soil moisture (MS_{max}, dMS_{min}), mean daily and minimum mean daily air temperature (dTA, dTA_{min}), and frost free days (FFD); 7.2% of total variance] and (C) both chemistry and climate (11% of total variance). The effects of year, spatial distance and shared effects (6.7% of total variance) were removed in each depicted model. Climate and litter chemistry factors were reduced by building the best 1-9 factor models for each subset (using 'leap' model selection for multivariate responses, Cerdeira et al. 2015) [62], selecting models where factors are all significant (using permutation test for CCA) and repeating with a joint set of the retained factors. The significance of final models was confirmed using a permutation test for CCA.
